# Controlling Inputter Variability in Vignette Studies Assessing Web-Based Symptom Checkers: Evaluation of Current Practice and Recommendations for Isolated Accuracy Metrics

**DOI:** 10.2196/49907

**Published:** 2024-05-31

**Authors:** András Meczner, Nathan Cohen, Aleem Qureshi, Maria Reza, Shailen Sutaria, Emily Blount, Zsolt Bagyura, Tamer Malak

**Affiliations:** 1 Healthily London United Kingdom; 2 Institute for Clinical Data Management Semmelweis University Budapest Hungary

**Keywords:** symptom checker, accuracy, vignette studies, variability, methods, triage, evaluation, vignette, performance, metrics, mobile phone

## Abstract

**Background:**

The rapid growth of web-based symptom checkers (SCs) is not matched by advances in quality assurance. Currently, there are no widely accepted criteria assessing SCs’ performance. Vignette studies are widely used to evaluate SCs, measuring the accuracy of outcome. Accuracy behaves as a composite metric as it is affected by a number of individual SC- and tester-dependent factors. In contrast to clinical studies, vignette studies have a small number of testers. Hence, measuring accuracy alone in vignette studies may not provide a reliable assessment of performance due to tester variability.

**Objective:**

This study aims to investigate the impact of tester variability on the accuracy of outcome of SCs, using clinical vignettes. It further aims to investigate the feasibility of measuring isolated aspects of performance.

**Methods:**

Healthily’s SC was assessed using 114 vignettes by 3 groups of 3 testers who processed vignettes with different instructions: free interpretation of vignettes (free testers), specified chief complaints (partially free testers), and specified chief complaints with strict instruction for answering additional symptoms (restricted testers). κ statistics were calculated to assess agreement of top outcome condition and recommended triage. Crude and adjusted accuracy was measured against a gold standard. Adjusted accuracy was calculated using only results of consultations identical to the vignette, following a review and selection process. A feasibility study for assessing symptom comprehension of SCs was performed using different variations of 51 chief complaints across 3 SCs.

**Results:**

Intertester agreement of most likely condition and triage was, respectively, 0.49 and 0.51 for the free tester group, 0.66 and 0.66 for the partially free group, and 0.72 and 0.71 for the restricted group. For the restricted group, accuracy ranged from 43.9% to 57% for individual testers, averaging 50.6% (SD 5.35%). Adjusted accuracy was 56.1%. Assessing symptom comprehension was feasible for all 3 SCs. Comprehension scores ranged from 52.9% and 68%.

**Conclusions:**

We demonstrated that by improving standardization of the vignette testing process, there is a significant improvement in the agreement of outcome between testers. However, significant variability remained due to uncontrollable tester-dependent factors, reflected by varying outcome accuracy. Tester-dependent factors, combined with a small number of testers, limit the reliability and generalizability of outcome accuracy when used as a composite measure in vignette studies. Measuring and reporting different aspects of SC performance in isolation provides a more reliable assessment of SC performance. We developed an adjusted accuracy measure using a review and selection process to assess data algorithm quality. In addition, we demonstrated that symptom comprehension with different input methods can be feasibly compared. Future studies reporting accuracy need to apply vignette testing standardization and isolated metrics.

## Introduction

### Background

Web-based symptom checkers (SCs) are tools for lay people to assess their symptoms using smartphones, tablets, and computers, providing possible conditions or triage or both. SC triage determines if there is a need to seek medical advice and its degree of urgency. The number of SCs is growing rapidly with generative artificial intelligence (AI) models gaining widespread popularity and being evaluated for their potential use as SCs [1]. Regulatory bodies often categorize SC as low-risk medical devices requiring only self-certification before introduction in the open market [2]. Evaluation of SCs is crucial; incorrect advice on triage or condition outcomes may result in patient harm [3]. Currently, there are no widely accepted criteria to assess their performance.

The 2 most frequently used methods of assessing SC performance are clinical studies and simulated patient studies via vignettes [2].

The Food and Drugs Administration and European Union Medical Device Regulation strongly recommend studies involving real patients to evaluate the performance of any medical device before its introduction in the market [4,5]. However, clinical trials have limitations. The cost can be prohibitive [2] and often only small population samples are feasible, limiting generalizability. In addition, these studies are conducted in a health care setting [6,7] and may not represent the population of individuals using an SC, who often use them before deciding whether to seek medical advice. Finally, the need for repeated assessments with every iteration of development might limit the feasibility of using real-patient studies.

Therefore, vignette studies have gained popularity. Vignettes are clinical scenarios described in a few sentences in accordance with a specific disease or differential list of diseases. Vignette studies offer the opportunity to assess multiple SCs simultaneously with a range of diseases and patient presentations within a single study at relatively lower cost [8]. However, they have several ontological, methodological, and epistemological limitations [9]. These include issues concerning the study of a rapidly evolving field; the creation of appropriate vignettes that are representative of real cases, populations, and health care settings; definition of gold standards; and trade-off between simple and complex or ambiguous cases [9-12].

### Accuracy as an Outcome

#### Overview

Most studies evaluate SC performance by measuring the accuracy of most likely conditions (single or multiple) or triage outcome determined by health care professionals [13-15]. Accuracy of the outcome behaves as a composite measure as it is affected by a number of individual SC- and tester-dependent factors. Some of these tester-dependent factors are only specific to the vignette methodology.

#### SC-Dependent Factors

SCs vary in their database, algorithm, symptom expression, and comprehension of chief complaints (ie, initial symptoms of a consultation mimicking a real patient’s presenting complaints) [16-18]. Any of these may impact the levels of outcome accuracy.

#### General Tester-Dependent Factors

Both real-life users and testers are likely to vary in their ability to express their symptoms and to comprehend the ones offered by the SC [19]. Misinterpretation of symptoms offered by the SC during the consultation may result in the addition of symptoms the user does not have and neglect the ones that they do have in reality. Moreover, how users or testers express the chief complaints can influence the outcomes. Even an SC with the best comprehension abilities will fail if the user expresses their symptoms incorrectly.

#### Tester-Dependent Factors Specific to Vignette Studies

Some of the general limitations of vignette studies are reported more often in the literature and have been mentioned in previous sections. However, some other tester-dependent limitations specific to vignette methodology measuring the accuracy of outcome are less explored.

Several vignette studies use vignettes that do not define chief complaints [13,20], leaving it to the discretion of the individual testers to select and interpret them. Appropriate determination of the chief complaints is an important aspect. A number of SCs provide different weighting to them compared to symptoms selected during the consultation. Thus, testers by selection of chief complaints can substantially influence the final outcome, including limiting the potential differentials and subsequent symptoms asked during the consultation [21]. Other studies have opted for an approach, where the chief complaints are prescribed [14,22]. A limitation of most of these studies is that the number of chief complaints was reduced to a single symptom. Therefore, these studies may not represent the complexity of patients presenting with multiple or ill-defined symptoms.

The other limitation of testers in vignette studies is caused by the extra step whereby testers have to understand and translate the vignettes for the SC. Their abilities to convey the intention of the vignette can influence the outcome.

### Hypothesis and Aims

We hypothesize that vignette studies measuring SC’s accuracy of outcome as a composite metric is not a reliable method to determine the performance and their result might not be generalizable to a wider population. In clinical studies with a large, diverse population, a wide range of symptom expression and comprehension ability is represented, which is similar to real life. However, in vignette studies, with few testers, the testers’ ability to input the chief complaints as intended or to select the right symptoms throughout the consultation can unduly influence results despite using the same SCs and clinical vignettes. This is why some studies have reported significant tester variability [20].

The primary aim of this study was to investigate the impact of tester variability on the accuracy of outcome of SCs, using clinical vignettes. The secondary aim was to identify the methodology to measure isolated aspects of SC performance using clinical vignettes. We investigated the following two isolated aspects: (1) SC data and algorithm quality using adjusted accuracy metrics and (2) SC symptom comprehension.

## Methods

### Clinical Vignettes

The Royal College of General Practitioners in the United Kingdom produced 139 vignettes to evaluate an SC in a benchmarking study conducted by the Self-Care Academic Research Unit of Imperial College London [20]. A total of 25 vignettes were excluded because they described asymptomatic individuals or individuals with long-term conditions that had already been diagnosed or because they lacked agreed-upon outcomes. The remaining 114 vignettes were used (minor alterations were performed to enhance the clarity of symptoms in 5 vignettes; Table S1 in Multimedia Appendix 1).

### Testers

We included 7 testers who were not employed by Healthily and were remunerated on an hourly basis. They were blinded to the real aims of the study. Nonmedical testers, who were either in university or had completed their education, were used for the inter- and intra-agreement analyses. The tester analyzing the comprehension of the SC was a medical student.

### Inter- and Intratester Agreement

#### Overview

This study consisted of 3 experimental phases to analyze the difference between inter- and intra-agreement of testers with differing testing instructions. Consultations were conducted by 3 testers using clinical vignettes with different instructions for each phase. The different instructions given for the different phases can be found in Multimedia Appendix 2.

Each tester was given the set of vignettes to input the data points (ie, gender, age, duration of complaints, symptoms present, and comorbidities) into Healthily AI Smart Symptom Checker (Healthily SC) via the website application [23]. The testers recorded every data point of the consultation. The 3 test phases were conducted using the OSC versions from June 2022, February 2023, and April 2023, respectively. The interagreement of testers was investigated in each phase. For each vignette, the most likely outcome condition provided by the SC was translated to a numerical value and compared to assess agreement. A score of 0 was assigned to cases where no condition was found as an outcome. Triage was evaluated using a rating system comprising 9 categories (ie, no triage, see a physician, self-limiting, self-care, routine, urgent—within 48 hours, urgent—within 12 hours, emergency and accident department and emergency ambulance).

#### Phase 1: Baseline

In phase 1, testers were free to select the chief complaints from the vignettes using their own interpretation without any imposed restrictions (*free tester group*). Intertester agreement was calculated for inputted chief complaints and consultation outcome. In addition, intratester agreement was also calculated following a second round where the same testers repeated vignette inputs with the same instructions approximately 6 months later.

For the agreement of input three investigators compared the chief complaints of the testers for similarity in 2 aspects: the exact wording and clinical concept. Exact wording was defined when 2 input symptom matched word for word. Clinical concept was defined when clinical symptoms matched using synonyms.

#### Phase 2: Restriction of Only Chief Complaints

Phase 2 investigated the effect of the chief complaints on the outcome by predefining the chief complaints for each vignette (partially free tester group). These chief complaints were prepared by 3 medical qualified doctors selecting single or multiple symptoms from each vignette and transforming them into unambiguous plain English terms. Agreement for most likely condition and triage was calculated.

#### Phase 3: Restriction of All Symptoms

Phase 3 involved further restrictions to investigate the effect of the other vignette study–specific tester-dependent factor: symptom translation from the vignette for the SC. Along with the prescribed chief complaints, strict instructions were given to each tester regarding the additional symptoms offered by the SCs (restricted tester group). Each tester was instructed to decline every symptom that was not specifically written in the vignette except for those that were synonyms or part of a wider logical category. For example, if the vignette described “pain in knee,” the tester would select “pain in leg” but decline a symptom called “pain on walking,” unless it is specified in the vignette explicitly. Agreement for the most likely condition and triage was calculated.

### Accuracy

The effect of variability between testers was evaluated by investigating the difference in the accuracy between the testers and groups. Accuracy was measured as the most likely condition outcome of the consultation matching the imperial gold standard [20]. The accuracy for each tester was calculated and then averaged for an overall measure in each group.

### Review and Selection Process for the Development of an Isolated Metric

In the restricted group, the presence of residual variability between tester outcomes and a difference between the highest and lowest performing testers even with the restrictions triggered the implementation of a review and selection process. The aim was to understand the source of variability and to develop an adjusted accuracy measure to assess data and algorithm quality in isolation.

Cases of the restricted group were reviewed by 2 researchers. First, for each case, all the data points from the consultation report for each tester, including symptom duration, comorbidities, age, gender, and symptoms that were selected or declined, were compared to the original vignette. Discrepancies from the initial vignettes were analyzed (eg, missing symptoms, addition of symptoms not described in the vignette, misinterpretation of symptoms, and selection of incorrect duration). Second, we selected tester consultations where no discrepancy was present. Descriptive analysis was used to describe the proportion of cases where consultations were fully identical to the vignettes stratified by matching outcomes between testers. Finally, an adjusted accuracy was calculated by (1) excluding cases where none of the 3 tester’s consultations were identical to the vignette’s data points (Accuracy_Excluded_) and (2) including results of a retested consultation for those cases correcting the discrepancies between the vignette and the initially performed consultations (Accuracy_Retested_).

### SC Comprehension

A feasibility study was conducted in March 2023 to evaluate the symptom comprehension of SCs in isolation. We defined symptom comprehension as the ability of the SC to understand the intended chief complaints inputted by the tester.

A total of 29 (25.4%) vignettes were randomly selected from the 114 vignettes previously used. The chief complaints extracted during phase 2 were used. Furthermore, 3 synonyms of these chief complaints were created for a natural language processing (NLP) input method and 3 for a drop-down menu method (refer to the list of all symptoms in Table S2 in Multimedia Appendix 1). NLP is a combination of methods based on linguistics, computer science, and AI, allowing computers to interpret and comprehend human language from written text [24,25].

A single tester inputted all the symptoms across 3 SCs: 1 using free text NLP (Healthily) and 2 using drop-down or search menus (Ada Health App [by Ada Health GmbH] and Infermedica triage [by Infermedica]) [23,26,27]. In one of the latter SCs, only 1 symptom can be inputted at a time, while in the other one, multiple symptoms can be inputted at once. The NLP SC has a drop-down option in the subsequent step after the NLP input, which the tester was allowed to use.

The tester had strict instructions to input the exact wording for the chief complaints and documented the response of the SCs.

Both NLP and drop-down menu inputs were scored in a similar fashion. A score of 2 was given when the exact symptom was detected or the same meaning was conveyed. For example, if the input was “lower tummy pain” and this was translated to “lower abdominal pain,” it scored 2. For extraction or offering of a wider logical category symptom, which would later allow other symptoms to be asked about later, a score of 1 was assigned. For example, if “lower tummy pain” was inputted and “abdominal pain” was extracted, it would score 1. An incorrect symptom, for example, if “passing too much urine” was the chief complaints and only “burning on passing urine” was shown, would score 0. The final scores were turned into probabilities.

### Statistical Analysis

Fleiss and Cohen κ using Stata 13 SE (StataCorp) and Package Kappaetc and proportion of agreement were measured [28,29]. The κ value was classified as per Landis and Koch [30] into the following groups: 0.00 and 0.20 as “slight,” between 0.21 and 0.40 as “fair,” between 0.41 and 0.60 as “moderate,” between 0.61 and 0.80 as “substantial,” and between 0.81 and 1.00 as “almost perfect.”

Descriptive statistics were used to analyze the accuracy of the most likely condition and the performance of symptom comprehension. Inferential statistics was used to compare accuracy between tester groups (ANOVA for all 3 groups and student 2-tailed *t* test to compare partially free and restrictive groups independently to the free group).

### Ethical Considerations

The study was reviewed by the Semmelweis University’s Institutional Review Board and determined to be Institutional Review Board exempt. The study did not involve the recruitment of patients or participants or use patient-identifiable data. Healthily holds the rights to the data. The testers were contracted to perform the task and were renumerated on an hourly basis (£15/hour; US $19).

## Results

### Clinical Vignettes

Characteristics of vignettes including demographics, duration of symptoms, expected triage categories, and the medical domains can be found in the Multimedia Appendix 3.

### Testers

Testers were aged between 21 and 39 years, and 33% (2/6) were female. More details on their demographics and which phase they participated in can be found in Multimedia Appendix 4.

### Inter- and Intratester Agreement

Intertester agreement (κ) for the most likely condition (κ=0.49) and triage (κ=0.51) were “moderate” in the free tester group and “substantial” in both the partially free group (κ=0.66 for both the most likely condition and triage) and the restricted group (κ=0.72 for the most likely condition and κ=0.71 for triage). The difference in κ between the restricted and free group is likely significant as the CIs do not overlap. The number of vignettes that had full agreement for the most likely condition increased from 36 to 73 and increased from 48 to 73 for triage, comparing the free tester group to the restricted group. The highest agreement was in the restricted group, with 63.2% full agreement, followed by 27.2% for partial agreement and 9.6% for no agreement for the most likely condition, and 64%, 34.2% and 1.8%, respectively, for triage (Table 1).

Intratester agreement showed κ ranging from 0.41 to 0.50 for the most likely condition and 0.44 to 0.53 for triage. The proportion of agreement ranged from 46.5% to 50.9% for the most likely condition and 50.0% to 59.6% for triage (Table 2).

Negative agreement of the chief complaints selected for the free tester group was demonstrated with κ=−0.4 (95% CI −0.44 to −0.35) for comparing the exact wording and −0.19 (95% CI −0.25 to −0.13) for comparing medical concepts (Table 3). There was no variability (κ=1) for input in the partially free or the restricted tester groups as these were prescribed.

κ intratester agreement for the chief complaints in the free tester group ranged from −0.55 to −0.78 when comparing exact wording and −0.32 to −0.51 when comparing medical concepts (Table 4).

**Table 1:** Intertester agreement for the most likely condition and triage outcome in the 3 tester groups with different testing instructions (N=114).

		Fleiss κ (95% CI)	*P* value	Full agreement, n (%)	Partial agreement, n (%)	No agreement, n (%)
**Most likely condition**						
	Free group	0.49 (0.42-0.56)	<.001	36 (31.6)	58 (50.9)	20 (17.5)
	Partially free group	0.66 (0.58-0.73)	<.001	62 (54.4)	40 (35.1)	12 (10.5)
	Restricted group	0.72 (0.65-0.79)	<.001	73 (64)	32 (28.1)	9 (7.9)
**Triage**						
	Free group	0.51 (0.44-0.59)	<.001	48 (42.1)	56 (49.1)	10 (8.8)
	Partially free group	0.66 (0.58-0.73)	<.001	68 (59.6)	37 (32.5)	9 (7.9)
	Restricted group	0.71 (0.64-0.78)	<.001	73 (64.0)	39 (34.2)	2 (1.8)

**Table 2:** Intratester agreement for the most likely condition and triage outcome of each of the 3 testers with vignette inputs repeated under the same instructions 6 months apart (N=114).

		κ (95% CI)	*P* value	Matches, n (%)
**Most likely condition**				
	Tester 1	0.50 (0.40-0.60)	<.001	58 (50.9)
	Tester 2	0.44 (0.35-0.54)	<.001	55 (48.2)
	Tester 3	0.41 (0.31-0.50)	<.001	53 (46.5)
**Triage**				
	Tester 1	0.44 (0.34-0.55)	<.001	60 (52.6)
	Tester 2	0.53 (0.40-0.63)	<.001	68 (59.6)
	Tester 3	0.44 (0.33-0.53)	<.001	57 (50)

**Table 3:** Intertester agreement for the chief complaints for the free tester group comparing the exact wording and medical concepts (N=114).

	κ (95% CI)	*P* value	Full agreement, n (%)	Partial agreement, n (%)	No agreement, n (%)
Exact wording	−0.40 (−0.44 to −0.35)	<.001	4 (3.5)	16 (14)	94 (82.5)
Medical concept	−0.19 (−0.25 to −0.13)	<.001	21 (18.4)	40 (35.1)	53 (46.5)

**Table 4:** Intratester agreement for the chief complaints for each of the 3 testers comparing the exact wording and medical concepts with vignette inputs repeated under the same instructions 6 months apart (N=114).

		Fleiss κ (95% CI)	*P* value	Matches, n (%)
**Exact wording**				
	Tester 1	−0.55 (−0.65 to −0.45)	<.001	31 (27.2)
	Tester 2	−0.74 (−0.84 to −0.64)	<.001	34 (29.8)
	Tester 3	−0.78 (−0.88 to −0.68)	<.001	14 (12.3)
**Concept**				
	Tester 1	−0.32 (−0.40 to −0.24)	<.001	58 (50.9)
	Tester 2	−0.48 (−0.58 to −0.38)	<.001	65 (57)
	Tester 3	−0.51 (−0.61 to −0.41)	<.001	37 (32.5)

### Accuracy

Accuracy of the 3 individual testers for the most likely condition was 43.9%, 50.9% and 57%, respectively, with an average of 50.6% (SD 5.35%) in the restricted group. Detailed individual results of all testers are provided in Multimedia Appendix 5.

When comparing the accuracy of the 3 different groups, there was no significant difference (*P*=.13). There was a 5% (*P*=.48) difference in average accuracy between the free and restrictive groups. There was a 10.2% (*P*=.05) difference in average accuracy between the free and partially free groups.

### Review and Selection Process for the Development of an Isolated Metric

Of the 114 vignettes in the restricted group, 51 (44.7%) had a discrepancy between testers for either triage or for the top diagnosis or both. In 55.3% (63/114) of the vignettes, they all received the same most likely condition and triage. For the cases where the outcome among all testers matched, only 3.5% (4/114) bore no perfect resemblance to the vignette’s data points from any of the testers (Table 5). In 3 of these cases, the vignettes were ambiguous. Reviewing the consultations where the testers did not agree on the most likely condition or triage revealed that in 9.6% (11/114) of the cases, none of the tester’s consultation was perfectly identical to the vignette. Overall, there were only 30.7% (35/114) of cases where the consultation of all 3 testers were identical to the data points of the vignette. In 56.2% (64/114) of the cases, 1 or 2 tester’s consultation was identical to the initial vignettes. In 12.3% (15/114) of the cases, none of the testers’ consultations were identical to the vignettes (Table 5).

Analysis of the causes for discrepancy between the vignettes and the completed consultations of the restricted group can be found in Multimedia Appendix 6.

The average accuracy for all testers was 50.6% (SD 5.35%) in the restricted group. If the accuracy was expanded to count the result for each case from the tester or testers who met the gold standard increased to 63.2%. Adjusted accuracy excluding cases where no consultation of any of the 3 testers were identical to the vignette’s data points (Accuracy_Excluded_), the most likely condition accuracy was 55.6%. When an adjusted accuracy was calculated with these cases retested (Accuracy_Retested_), the accuracy was similar at 56.1% (Table 6).

**Table 5:** Number of cases where the testers’ consultation was identical to the vignette categorized by the tester’s agreement in outcome (both triage and condition outcome; N=114).

	0 consultation identical to the vignette, n (%)	1 consultation identical to the vignette, n (%)	2 consultations identical to the vignette, n (%)	All 3 consultations identical to the vignette, n (%)	Total, n (%)
2 or no testers match each other in outcome	11 (9.6)	17 (14.9)	23 (20.2)	0 (0)	51 (44.7)
All testers match each other in outcome	4 (3.5)	10 (8.8)	14 (12.3)	35 (30.7)	63 (55.3)
Total	15 (13.1)	27 (23.7)	37 (32.5)	35 (30.7)	114 (100)

**Table 6:** Most likely condition accuracy in the restricted group: average of 3 testers, accuracy if counted as outcome condition met the gold standard from any 1 of the testers, excluding cases where none of the consultations were identical the vignette’s data points (Accuracy_Excluded_), and if those cases were retested (Accuracy_Retested_).

	Accuracy of outcome condition (%)
Average of 3 testers (SD)	50.6 (5.35)
If any tester met the gold standard	63.2
Accuracy_Excluded_	55.6
Accuracy_Retested_	56.1

### SC Comprehension

The accuracy of the comprehension of chief complaints was easily measurable on both NLP SCs and those that use drop-down symptom input methods. In total, 51 symptoms were assessed (maximum achievable score of 306), covering 29 vignettes (maximum achievable score of 174). The percentage of symptoms understood ranged from 52.9% (162/306) to 68% (208/306) when assessed as stand-alone symptoms and ranged from 55.7% (97/174) to 64.4% (112/174) when symptoms were grouped by their respective vignettes (Table 7).

**Table 7:** Percentage of inputs understood by different symptom checkers per individual symptoms (51 symptoms, N=306) and individual symptoms grouped per vignette (29 vignettes; N=174).

	Results per individual symptom (n=306), n (%)	Results per vignette (n=174), n (%)
Symptom checker 1	208 (68)	112 (64.4)
Symptom checker 2	186 (60.8)	108 (62.1)
Symptom checker 3	162 (52.9)	97 (55.7)

## Discussion

### Principal Findings

We demonstrated significant variations in the agreement of outcome and chief complaints input between different testers of SCs using the same vignettes and when repeated by the same tester over time. Variability between the testers was significantly reduced by restricting testers on the selection of chief complaints and additional symptoms. This demonstrated that tester-dependent factors specific to vignette methodology are partly responsible for the variation and they can be reduced using our restrictive methods. However, even when controlled, significant variability in agreement between testers remained. The lack of agreement resulted in substantial differences in accuracy of condition outcome within the restricted group. The residual variability between restricted testers suggested that general tester-dependent factors and potentially some of the remaining vignette methodology–dependent factors play an important role in determining the outcome and consistency between testers. Studies with a large number of participants, such as clinical trials, can use outcome accuracy as a composite measure accounting for the tester-dependent factors. However, in studies with a small number of testers, such as vignette studies, tester-dependent factors can disproportionately influence the results. Therefore, outcome accuracy as a composite metric in such studies is at risk of reduced reliability and generalizability to a wider population.

Therefore, we established isolated measures of performance. We developed a review and selection process to ensure that only the results of consultations identical to the vignettes are used to calculate an adjusted accuracy (Accuracy_Excluded_ or Accuracy_Retested_). Using our adjusted accuracy, we were able to measure an isolated reflection of data and algorithm quality in vignette studies.

Another aspect of SC performance is symptom comprehension, which, as we demonstrated, is feasible to measure in isolation across a range of input methods.

### Comparison to Previous Works

No study to date has reported (1) the intratester agreement assessing the variability of chief complaints interpretation and selection, (2) the relationship between symptom input and outcome, or (3) the impact of tester variability on outcome.

Furthermore, most vignette studies do not report the intertester variability of outcome. One possible reason for the lack of data on intertester variability is that many studies have only used 1 sole tester for each test case [14,22,31,32]. However, as our study demonstrated, individual testers inconsistently input vignettes.

The degree of agreement in outcome for our free tester group was comparable to the study by El-Osta et al [20] where the same set of vignettes were used with free testers. However, it was lower compared to the study by Semigran et al [13], which had an agreement of 0.9. This may be explained by a lower number of vignettes in the study by Semigran et al [13], with only a sample of vignettes undergoing assessment for variability. Shen et al [15] also reported an agreement (0.74) higher than our free tester group but similar to our restricted group. However, κ value was calculated against the gold standard condition being in the top 3 outcome conditions of the SC [15], whereas we evaluated whether the testers had an exact match with each other for the top outcome condition. Most studies do not report on the instructions given to testers in answering the questions of the SC during consultation. The high agreement in some studies could be explained by the vignettes being potentially inputted with similar instruction to our restricted group.

Patients with the exact same symptoms but with different chief complaints drive the thought process of doctors [33,34]. Therefore, treating them as the exact same cases for the assessment of SCs is unfair and can result in different outcomes, as demonstrated in our study. Investigators should always assign chief complaints to vignettes with a mix of single and multiple symptoms to mimic real-patient presentation. We have found only 1 peer reviewed paper that provided cases with multiple chief complaints [16]. In contrast to vignettes that have the exact same symptoms but different chief complaints, vignettes that only differ in the way the chief complaints is expressed should be considered the same case. However, different expressions of the same symptom can and need to be assessed with a large number of cases because there are multiple synonyms or ways of expressing the same symptom, as demonstrated in this study. For example, abdominal pain may be referred to as “pain in the abdomen” or “tummy ache.” Some SCs may understand one of these terms but not others. Interestingly, the importance of assessing comprehension was also mentioned in the preprint paper by Kopka et al [12] published just before the submission of this paper.

There are multiple methods of assessing NLP [35], including good examples in the medical field [36]. However, only a few methods exist assessing drop-down menus and even less to compare NLP versus drop-down menu. Furthermore, NLP evaluations use metrics such as precision, recall, and F_1_ values that require false positives for the calculations. An SC that allows a user to correct errors in NLP symptom comprehension complicates the evaluation by allowing the user to remove false positives, while for false negatives, this process brings a drop-down menu into an otherwise NLP-driven symptom comprehension process. Hence, we used an intrinsic evaluation method with a very simple scoring system. Although 1 SC had a higher comprehension accuracy than the other 2, we do not consider it to be truly superior as this is a feasibility study.

### Relationship Between Agreement and Accuracy

Despite prescribing the chief complaint(s) in a vignette and restricting additional symptoms during the OSC-user interaction, which are important steps, they are still not sufficient to eliminate all variability among testers in outcomes. The consequences of this residual variability can influence accuracy. When comparing results between the tester groups, the difference for intertester agreement was significant between the free group and the other 2 groups (CIs do not overlap). A statistically significant difference in outcome condition accuracy was observed only between the free and partially free groups. However, significance has to be evaluated with caution in view of the small sample size, and the individual results might hold more lessons. They demonstrate within the restricted group that even with only 3 testers and a relatively good agreement, the accuracy difference between the “highest” and “lowest” performing testers can remain relatively high with 13%. This may also be one of the reasons why systematic reviews on accuracy of SCs show a marked spread of results when comparing individual vignette studies or both clinical and vignette studies [2,37-39]. From a detailed review of the tester consultations, accuracy does not clearly correlate with adherence to vignettes. Discrepancies from the vignettes occurred for different cases and for different symptoms; hence, the consequences and the impact on accuracy were varied. Furthermore, even if their outcome was in line with the gold standard, this did not always translate as a spotless representation of the vignette during the consultation.

Therefore, we believe that using adjusted accuracy through a review and selection process is an essential step in studies aiming to measure accuracy reliably. In the restricted phase of our study, there were 15 cases where all testers’ consultation had discrepancies to the vignettes. For this isolated metric, those cases with discrepancies need to either be excluded from analysis or repeated, as was conducted in our study. As shown in our study, only 3.5% (4/114) of cases were in groups where all testers agreed on both the most likely condition and triage. Therefore, we believe that in a real benchmarking exercise, it would be acceptable to only review the cases where the testers have a discrepancy in the most likely condition or triage.

Analyzing the consultation reports revealed that the causes for the discrepancies from the original vignette resulting in the varying outcome between testers are more multifactorial than what we assumed before the study. Some of these causes have implications for future vignette studies. Human error seems to be an important tester-dependent factor. Testers of vignettes may be inherently more prone to errors compared to real patients in studies as they do not report on their own symptoms. The fact that even in the restricted group this was a significant factor influencing outcome suggests that simply increasing the number of testers and applying restrictions would most likely not fully resolve those vignette methodology related limitations like our review and selection process.

Some of the discrepancies were due to the incompatibility between how the Healthily SC works and the instruction given to the testers. For example, in a situation where a user has already declared that they have a cough, they were required to select either a dry or productive cough as there were no “none of them” option. In such a scenario, testers were unable to adhere to restrictive instructions because the vignettes did not specify whether the cough was dry or productive. This raises the point that researchers conducting benchmarking studies should familiarize themselves with the SCs they are evaluating. Our recommended instructions following the takeaway points from this study and the detailed causes for discrepancies can be found in Multimedia Appendices 6 and 7.

Some other discrepancies were proven to be the consequence of a few ambiguously phrased or incomplete vignettes that can lead to the misinterpretation of symptoms. One of the vignettes specified “multiple sexual partners” but did not specify whether protection was used. If testers were allowed to decide the answer, the test results would become tester dependent and unreliable. Hence, strict instructions are important, even if that might cause some cases to not complete the consultation process. With the review and selection process, these vignettes can either be excluded from the analysis or retested after expanding their information content according to the anticipated answer from a real patient. In the case described above in this paragraph, all our testers confirmed unprotected sexual encounters, and they were all scored as not following the vignette. However, the vignette was then corrected and repeated.

It is impossible to write vignettes that anticipate every question an SC might ask that a real patient would be able to answer. Nevertheless, the vignette creation process has room for improvement. For example, there is a difference between the “main” symptoms and the characteristics of an already established symptom. Most patients would be expected to know whether they had unprotected sexual intercourse; however, some patients may struggle to describe whether the abdominal pain is at the top or middle of their abdomen. Another lesson is that the more detailed a symptom is, the less room it leaves for misinterpretation. Creators could always specify a symptom in as much detail as a real user could; therefore, rather than using the words “rash on leg,” the vignette could state “rash on leg spreading from thigh to knee.” Further research and guidelines are needed to establish how to write suitable vignettes specifically for SCs.

### Limitations

This study has several limitations. We have used only 3 testers for each variation of the study, which could have influenced the degree of agreement.

The testers who assessed the agreement between the outcomes were lay people. Previous studies have suggested that health care professionals are more reliable testers, but lay testers are closer to the real user base of SCs; hence, following the recommendation of Painter et al [10], we opted for the latter approach to assess outcome agreement [40,41]. In contrast, we felt that there is no significant difference between lay or medical testers for assessing comprehension accuracy, as these symptoms were entirely prescribed; hence, a medical student was assigned the task.

Another possible limitation of our study was that only 1 SC was used with only 1 set of vignettes. However, previously, we explored using an independent and smaller vignette set (51 vignettes) collated from 5 different sources and applied these on 3 different SCs using 2 testers. For each SC, the agreement between the 2 testers was <50%, suggesting that the results are independent of SCs and vignettes (Sutaria, S, unpublished data, June 2023).

Furthermore, we used interpretation of κ values by Landis and Koch [30] who originally developed it for use with only 2 annotators.

Only the most likely condition was considered in our study for the sake of simplicity when calculating agreement and accuracy. Assessing all the outcome conditions from a consultation may have potentially improved the accuracy results. However, the aim was to demonstrate the effect of variability on the accuracy and the importance of using a reliable methodology during the study and not to establish an accuracy score for the Healthily SC. Any full study assessing comprehension conducted by Healthily researchers could bias the results. Therefore, a simple methodology was used to score the results to test the feasibility rather than aiming to determine a true accuracy.

We used a small number of chief complaints and SCs for assessing comprehension accuracy prepared by the researchers. Using focus groups with lay people and larger numbers may be a more appropriate approach in future studies. However, the aim was to assess the feasibility of conducting such an analysis. As the study was financed by an SC company and the investigators are employees of the company, the authors felt that it would be biased to run a real assessment publishing a verdict on the accuracy of the comprehension of any SC.

### Conclusions and Recommendations

The authors of vignette studies should understand that they are unable to account for important variables when measuring accuracy using vignettes in the same manner as clinical studies. They should instead aim to measure isolated aspects of accuracy to judge the performance of an SC.

We recommend the following:

- Cautious comparison of triage and condition outcome accuracy in clinical trials against vignette studies
- Measuring adjusted accuracy (Accuracy_Excluded_/ Accuracy_Retested_) in vignette studies to assess the algorithm and data quality used by the model only through the following:
  Using vignettes with selected chief complaints that cover a variation of single and multiple symptoms that are clearly and plainly described
  Giving clear instructions to testers on not selecting any symptom that is not present in the vignette unless it is a synonym or a bigger category of a symptom described in the vignette (refer to the recommendation on instructions in Multimedia Appendix 7)
  Using multiple testers to input each vignette
  Comparing consultations to the vignette in cases where there is a discrepancy in the outcome between testers. This should be performed blindly without prior knowledge of the expected gold standard. Only consultations that accurately represent the vignette’s data points should be included for the evaluation. Exclusion or repetition of vignettes might be required. Vignettes might need adaptation or correction before repetition. Testers might need to draw attention to the misinterpretation of symptoms and human errors before retesting.
- Measuring and reporting on comprehension of the SCs

Further research is needed for the following:

- To develop methodologies to measure other aspects of performance such as the SC’s ability to express symptoms in an understandable manner during the consultation
- To develop methodologies and guidelines for creating vignettes specifically with the purpose of assessing SCs
- To explore the evaluation and comparison of other, different input methods

## Appendix

Multimedia Appendix 1 Test cases.

Multimedia Appendix 2 Instructions given to the testers for assessing intertester agreement.

Multimedia Appendix 3 Characteristics of the vignettes.

Multimedia Appendix 4 Characteristics of the testers.

Multimedia Appendix 5 Individual accuracy results of the testers in the different phases and the averages of each group.

Multimedia Appendix 6 Causes for discrepancy between the vignette and consultations.

Multimedia Appendix 7 Recommended instructions for future testers.

## Abbreviations

AI: artificial intelligence
NLP: natural language processing
SC: symptom checker

## Data Availability

The data sets generated during and analyzed during this study are published either in the main text or in the multimedia appendices. The exceptions are the clinical vignettes that are not publicly available, as this would compromise their use for continuous evaluation of Healthily’s web-based symptom checkers (SC). However, they are available from Healthily on reasonable request following approval for genuine scientific research. Requests that might compromise the use of the vignettes in further studies run by Healthily or whereby the vignettes might become publicly available will be denied. Data will be not shared with other companies working on SCs or companies that are competitors of Healthily. Every request will be evaluated on a case-by-case basis by Healthily.
