# Supplementary material for: Controlling Inputter Variability in Vignette Studies Assessing Web-Based Symptom Checkers: Evaluation of Current Practice and Recommendations for Isolated Accuracy Metrics
Source: JMIR Form Res. 2024 May 31;8:e49907. doi: 10.2196/49907 (PMC11179013; doi:10.2196/49907)
Supplement: Multimedia Appendix 1 [file formative_v8i1e49907_app1.docx]

### Table S1: Cases for assessment of tester inter- and intra-agreement

| **ID** | **Age** | **Gender** | **Duration** | **Chief Complaint(s)** | **OUTCOME 1** | **OUTCOME 2** | **OUTCOME 3** |
| --- | --- | --- | --- | --- | --- | --- | --- |
| **1** | 22 | Female | 6 weeks | Upper abdominal pain,  Abdominal pain worse after eating | GALLSTONES | REFLUX (Esophageal/Acid) | GASTRITIS (Gord) |
| **2** | 18 | Male | 1 month | Hearing voices | PSYCHOSIS (new onset, drug induced) | SCHIZOPHRENIA | ANXIETY |
| **4** | 18 | Female | 2 days | Lower abdominal pain,  Frequent urination | URINARY TRACT INFECTION ( | SEXUALLY TRANSMITTED INFECTION) | PELVIC INFLAMMATORY DISEASE |
| **5** | 40 | Female | 6 months | Feeling stressed and irritable,  Excessive worrying | ANXIETY DISORDER (Generalised) | DEPRESSION |  |
| **6** | 19 | Female | 1 day | Feeling faint,  Collapse | ANOREXIA (Nervosa) | ANAEMIA | TYPE 1 DIABETES |
| **7** | 80 | Female | 1 year | Memory loss | ALZHEIMER'S DISEASE (Dementia) | STROKE | DEPRESSION |
| **8** | 32 | Female | 3 months | Dry cracked skin of hands,  Obsessively washing hands,  Fear of dirt | OBSESSIVE COMPULSIVE DISORDER | DEPRESSION (Depressive disorder) | PHOBIC DISORDER (Germophobia) |
| **9** | 40 | Male | 2 months | Having nightmares,  Insomnia | PTSD | DEPRESSION | SOCIAL PHOBIA (Phobic anxiety) |
| **10** | 70 | Male | 6 weeks | Left hip pain,  Difficulty walking | OSTEOARTHRITIS | TROCHANTERIC (Greater Trochanteric bursitis/ Trochanteric bursitis) | MUSCLE SPRAIN |
| **11** | 30 | Male | 1 week | Left knee pain after exercise,  Left knee swelling after exercise | MENISCAL TEAR | LIGAMENT INJURY (Acute/ Knee) | FRACTURE OF THE KNEE BONE ( |
| **12** | 60 | Male | 1 month | Right heel pain after walking | BACK PAIN (Muscular/ lumbar/ mechanical) | RHEUMATOID ARTHRITIS) | FRACTURE OF THE HEEL BONE |
| **13** | 20 | Male | 1 week | Lower back pain after heavy lifting | BACK PAIN (Muscular/ lumbar/ mechanical) | HERNIATED LUMBAR DISC | KIDNEY INFECTION |
| **14** | 50 | Female | 1 month | Pain in both hands which is worse around the knuckles | OSTEOARTHRITIS | RHEUMATOID ARTHRITIS | GOUT |
| **15** | 75 | Male | 1 day | Severe right big toe pain,  Right big toe is red,  Right big toe pain came on suddenly | GOUT | ARTHRITIS (Reactive/ infective/ septic) | INFECTED JOINT |
| **16** | 19 | Male | 2 days | Left sided tongue pain | MOUTH ULCER | DENTAL PROBLEM (Abscess/ irritation from tooth) | CORONITIS |
| **18** | 52 | Male | 1 week | Painful urination | SEXUALLY TRANSMITTED INFECTION | URINARY TRACT INFECTION | THRUSH |
| **19** | 72 | Female | 2 days | One sided headache,  Unable to sleep due to headache | MIGRAINE | HEADACHE | GIANT CELL ARTERITIS |
| **20** | 18 | Female | 1 week | Swollen ankles,  Dark urine,  Frequent urination | NEPHROTIC SYNDROME | GLOMERULONEPHRITIS | URINARY TRACT INFECTION |
| **21** | 32 | Female | 1 month | Crampy abdominal pains,  Abdominal pain after eating dairy or gluten | IRRITABLE BOWEL SYNDROME | COELIAC DISEASE | ANAEMIA (Menstrual related) |
| **22** | 24 | Male | 4 weeks | Cough | ASTHMA (Occupational) | ALLERGY (Horses/ Allergic asthma) | POST VIRAL COUGH |
| **23** | 23 | Male | 2 weeks | Itchy red rash on arms,  Rash worse after exercise | URTICARIA (Acute/ Chronic/ Idiopathic/ Viral/ Allergy) | ALLERGY (Contact/ rash/ urticaria) |  |
| **24** | 17 | Female | 1 day | Recurrent nosebleeds,  Bruises on thigh,  Very pale | LEUKAEMIA (Acute/ Haematological malignancy) | SEPSIS | ANAEMIA |
| **25** | 34 | Male | 2 weeks | Sore neck glands,  Hot and sweating at night | LYMPHOMA | INFECTION (Unidentified viral) | GLANDULAR FEVER |
| **28** | 48 | Male | 1 week | Lower back pain, | MYELOMA | CANCER (Bowel) | STRESS AT WORK |
| **29** | 23 | Male | 1 day | Short of breath,  Fever of 38.2 degrees,  Red wound,  Hot wound | SEPSIS) | WOUND INFECTION | CHEST INFECTION |
| **31** | 61 | Female | 3 days | Cough,  Shivering | COPD (Exacerbation/ Underlying) | LUNG CANCER | PNEUMONIA |
| **32** | 26 | Female | 2 days | Painful throat,  Cough | SORE THROAT (Bacterial/ Viral/ Infective) | TONSILLITIS | GLANDULAR FEVER |
| **34** | 18 | Female | 2 days | Runny nose,  Watery eyes,  Sticky eyes | VIRAL CONJUNCTIVITIS | BACTERIAL CONJUNCTIVITIS | ALLERGY |
| **36** | 55 | Male | 1 month | Blood in urine | CANCER OF THE KIDNEY | KIDNEY STONE | GALL BLADDER STONE |
| **38** | 25 | Female | 2 weeks | Burning pain on urinating | SEXUALLY TRANSMITTED INFECTION (CHLAMYDIA/ GONORRHOEA) | URINARY TRACT INFECTION | BLADDER CALCULUS |
| **39** | 30 | Female | 1 week | Lower abdominal pain,  Fever of 39 degrees,  Chills,  Vomiting | PELVIC INFLAMMATORY DISEASE | SEXUALLY TRANSMITTED INFECTION (GONORRHOEA) | ECTOPIC PREGNANCY |
| **40** | 21 | Female | 5 weeks | Smelly, watery vaginal discharge | BACTERIAL VAGINOSIS | SEXUALLY TRANSMITTED INFECTION | VAGINAL CANDIDIASIS |
| **42** | 48 | Male | 1 week | Frequent urination,  Scrotum pain,  Shivering,  Fever | URINARY TRACT INFECTION | EPIDIDYMO-ORCHITIS (Epididymitis) | ACUTE PROSTATITIS |
| **43** | 21 | Female | 6 months | Pelvic pain before and after period | ENDOMETRIOSIS | FIBROIDS | OVULATION PAIN |
| **44** | 24 | Female | 6 months | Heavy periods,  Painful periods | FIBROIDS | MENORRHAGIA | PRIMARY DYSMENORRHOAE/ MENSTRUAL PAIN |
| **45** | 52 | Female | Hours | Upper abdominal pain,  Chest pain | HEART ATTACK (Myocardial infarction) | GASTRITIS | GALLSTONES |
| **46** | 85 | Male | Hours | Central abdominal pain,  Abdominal pain radiating to back and groin | ABDOMINAL AORTIC ANEURYSM RUPTURE | BOWEL PERFORATION | APPENDICITIS |
| **47** | 20 | Female | 1 week | Lower abdominal pain,  Thick vaginal discharge | PELVIC INFLAMMATORY DISEASE | SEXUALLY TRANSMITTED DISEASE | APPENDICITIS |
| **48** | 28 | Female | 1 day | Right sided abdominal pain,  Nausea,  Loss of appetite | ECTOPIC PREGNANCY | APPENDICITIS | MISCARRIAGE |
| **50** | 60 | Male | 1 month | Chest tightness,  Chest pain worse on exertion,  Chest pain radiating to neck | ANGINA (Pectoris) | GASTRIC REFLUX | PANIC ATTACK |
| **51** | 47 | Female | 1 week | Smelly vaginal discharge,  Frequent Periods,  Heavy periods | BACTERIAL VAGINOSIS | MENORRHAGIA | RETAINED TAMPON |
| **52** | 36 | Male | 2 months | Palpitations | TACHYCARDIA (Sinus) | ATRIAL FIBRILLATION (Cardiac rhythm abnormality) | EXCESS STIMULANTS |
| **53** | 24 | Female | Hours | Eyelid drooping,  Unable to smile | BELL'S PALSY | STROKE | MIGRAINE |
| **54** | 47 | Male | 1 week | Eye floaters | DETACHMENT (Posterior vitreous/ Retinal) | VITREOUS HAEMORRHAGE | MUSCULAR HOLE |
| **55** | 52 | Female | 6 weeks | Left heel pain which is worse in the morning | PLANTAR FASCIITIS | POORLY FITTING FOOTWEAR | SOFT TISSUE INJURY |
| **56** | 37 | Male | 4 months | Productive cough,  Weight loss | TUBERCULOSIS | LUNG CANCER | NON-RESPIRATORY CAUSE |
| **57** | 23 | Male | 3 months | Insomnia,  Stress and anxiety | ANXIETY | DEPRESSION | WORKPLACE STRESS |
| **58** | 65 | Female | 2 weeks | Palpitations | TACHYCARDIA (Supraventricular/ Sinus) | ECTOPIC VENTRICULAR BEATS | ATRIAL FIBRILLATION |
| **59** | 52 | Male | 2 months | Chest pain on exertion | ANGINA | INDIGESTION | PULMONARY EMBOLISM |
| **60** | 55 | Male | 20 minutes | Central chest pain,  GTN spray has not helped,  Chest pain radiates to jaw | MYOCARDIAL INFARCTION | ANGINA (unstable) | PULMONARY EMBOLISM |
| **62** | 79 | Female | 2 hours | Dizziness,  Palpitations,  Feeling faint | BRADYCARDIA | CARDIAC ARRHYTHMIA | VASOVAGAL SYNCOPE |
| **63** | 21 | Female | 2 weeks | Palpitations | ANXIETY | TACHYCARDIA | HYPERTHYROIDISM |
| **64** | 25 | Female | Hours | Sore throat | VIRAL SORE THROAT | TONSILLITIS (Bacterial) | PERITONSILLAR ABSCESS |
| **65** | 32 | Female | 4 months | Blocked nose on both sides,  Runny nose on both sides | RHINITIS (Chronic/ allergic) | STAPHYLOCOCCAL NASAL INFECTION | SINONASAL TUMOUR |
| **66** | 50 | Male | 1 month | Decreased hearing in right ear | EAR WAX | EAR INFECTION | ACOUSTIC NEUROMA |
| **67** | 29 | Female | Few days | Right ear pain,  Itchy ear | EAR INFECTION (Otitis externa/ otitis media) | RAMSAY HUNT SYNDROME | MENIERE'S DISEASE |
| **68** | 38 | Female | 3 months | Recurrent nose bleeds from both nostrils | INFECTION (Nasal) | SINONASAL TUMOUR | ACUTE SINUSITIS |
| **70** | 24 | Male | 6 weeks | Pain on outside of elbow | TENNIS ELBOW | MUSCLE STRAIN | BURSITIS |
| **71** | 39 | Female | 3 weeks | Lower left back pain | BACK PAIN (musculoskeletal/mechanical) | MUSCLE STRAIN (lumbar ligament/paravertebral muscle) | KIDNEY STONE (renal pathology) |
| **72** | 43 | Male | Few days | Right big toe pain,  Right big toe swollen,  Right big toe red | GOUT | INFECTION (big toe) | SYNOVITIS |
| **73** | 76 | Female | 6 months | Left knee pain that is worsening | OSTEOARTHRITIS (knee) | MENISCAL (tear/injury) | GOUT |
| **74** | 68 | Male | Few weeks | Shoulder pain,  Thigh pain | POLYMYALGIA RHEUMATICA | OSTEOARTHRITIS | FIBROMYALGIA |
| **75** | 67 | Male | 20 minutes | Neck pain,  Unable to move neck,  Recent injury | FRACTURED CERVICAL SPINE | SPINAL TUMOUR (bony metastases) | INTERVERTEBRAL DISC PROLAPSE |
| **76** | 28 | Male | 24 hours | Unable to pass urine | CAUDA EQUINA SYNDROME (spinal cord compression) | PROLAPSED DISC (intervertebral) | SPINAL STENOSIS |
| **77** | 29 | Male | 10 days | Dry cough | VIRAL UPPER RESPIRATORY TRACT INFECTION (cough/chest infection) | ASTHMA (airways narrowing) | PNEUMONIA |
| **80** | 18 | Female | 3 weeks | Shortness of breath,  Palpitations,  Sweating | ANXIETY (panic attack) | ASTHMA | CARDIAC ARRHYTHMIA (irregular heart rhythm) |
| **82** | 30 | Male | 24 hours | Right sided chest pain,  Shortness of breath | MUSCULOSKELETAL CHEST PAIN (costochondritis) (3/12) | Pneumothorax | PULMONARY EMBOLISM |
| **84** | 37 | Female | Few hours | Right sided facial drooping | BELL'S PALSY (facial nerve palsy) | RAMSAY HUNT SYNDROME (stroke/CVA) | TIA/MULTIPLE SCLEROSIS |
| **85** | 17 | Female | 1 day | Severe headache,  Left sided headache | MIGRAINE | STROKE | HAEMORRHAGE (acute brain/subarachnoid) |
| **87** | 34 | Female | 2 days | Sore red eye,  Sensation of foreign body in eye | CONJUNCTIVITIS | CORNEAL ABRASION (foreign body) | EPISCLERITIS/SCLERITIS |
| **88** | 42 | Male | 3 days | Worsening right sided facial drooping | BELL'S PALSY (facial nerve palsy) | STROKE (cva) | MYASTHENIA GRAVIS |
| **89** | 21 | Female | 1 day | Left eye is red,  Itchy eyes,  Eyelids stuck together | BACTERIAL CONJUNCTIVITIS | ALLERGIC CONJUNCTIVITIS | VIRAL CONJUNCTIVITIS |
| **90** | 45 | Female | 1 month | Right sided abdominal pain,  Intermittent abdominal pain,  Abdominal pain worse after eating | GALLSTONES (biliary colic) | STOMACH ULCER | PANCREATITIS |
| **91** | 19 | Female | 7 days | Frequent urination,  Burning pain on urination,  Lower abdominal pain | UTI (urine infection/urinary tract infection) | STI | DIABETES MELLITUS |
| **92** | 51 | Male | FFew hours | Upper abdominal pain,  Worsening upper abdominal pain,  Fever | ACUTE CHOLECYSTITIS | PANCREATITIS (acute) | GASTROENTERITIS |
| **93** | 69 | Male | 2 days | Pain on passing urine,  Lower right back pain,  Fever of 38.6 degrees | PYELONEPHRITIS (uti with pyelonephritis) | PROSTATITIS | RENAL STONES |
| **94** | 56 | Female | 5 weeks | Pins and needles in left hand,  Pins and needles in left hand worse at night,  Intermittent pain in left hand,  Pain in left hand worse at night | CARPAL TUNNEL SYNDROME | CERVICALGIA RADICULOPATHY (with nerve symptoms) | LATERAL EPICONDYLITIS |
| **95** | 47 | Female | Few hours | Right sided facial drooping | BELL'S PALSY (facial nerve palsy) | STROKE (cva) | RAMSAY HUNT SYNDROME |
| **96** | 17 | Female | 1 day | Severe headache,  Left sided headache,  Vomiting | MIGRAINE | STROKE (ischaemic/haemorrhagic) | BIH |
| **97** | 52 | Male | 2 months | Right hand tremor | PARKINSON'S DISEASE (parkinsonism) | (benign) ESSENTIAL TREMOR | ALCOHOL ABUSE |
| **98** | 27 | Female | 2 weeks | Daily headaches,  Frontal headaches | TENSION HEADACHE | MIGRAINE | STRESS |
| **99** | 21 | Male | Few hours | Collapse,  Twitching movement of arms and legs | (general tonic clinic) SEIZURE (/epilepsy) | FAINT (vaso-vegal collapse) | NON EPILEPTIC ATTACK |
| **101** | 74 | Male | Some months | Numbness of feet | SPINAL CORD COMPRESSION (cauda equina) | CAUDA EQUINA | TYPE 2 DIABETES |
| **104** | 55 | Male | 6 weeks | Lump in right side of neck | REACTIVE LYMPH NODE (lymphoma/solitary lymph node cancer) | PRIMARY HEAD AND NECK CANCER | BRANCHIAL CYST |
| **109** | 45 | Male | 3 weeks | Productive cough,  Blood in phlegm | PNEUMONIA | LUNG CANCER (carcinoma lung) | TB |
| **110** | 26 | Female | 4 days | Cough,  Sore throat,  Blocked nose | URTI (viral) | HEART FAILURE | PNEUMONIA |
| **111** | 68 | Male | 8 weeks | Coughing,  Coughing up blood | LUNG CANCER (carcinoma lung) | PNEUMONIA | TB |
| **112** | 22 | Male | 5 weeks | Coughing,  Coughing up blood,  Fever | TB | PNEUMONIA | LUNG CANCER |
| **113** | 45 | Male | 3 days | Big toe swollen,  Big toe red,  Big toe painful when walking | GOUT | PARONYCHIA (infected toe) | FRACTURE OF TOE |
| **114** | 18 | Female | 1 week | Bilateral wrist joint swelling,  Bilateral ankle joint swelling | ARTHRITIS (juvenile/seronegative/reactive) | RHEUMATOID ARTHRITIS | JOINT INFECTION |
| **115** | 37 | Female | 8 weeks | Multiple joint pain,  Multiple joint stiffness | SYSTEMIC LUPUS ERYTHEMATOSUS | RHEUMATOID ARTHRITIS | PSORIATIC ARTHROPATHY |
| **116** | 65 | Male | 1 day | Right knee swelling,  Right knee redness,  Right knee pain | ARTHRITIS (septic/acute infective) | GOUT | RHEUMATOID ARTHRITIS |
| **117** | 75 | Male | 3 months | Right hip pain | OSTEOARTHRITIS (hip) | BURSITIS (trochanteric) | GOUT |
| **118** | 69 | Female | 3 weeks | Pain in neck,  Pain in shoulders,  Pain in hips | POLYMYALGIA RHEUMATICA | RHEUMATOID ARTHRITIS | SYSTEMIC LUPUS ERYTHEMATOSUS |
| **119** | 26 | Male | 3 days | Right sided abdominal pain,  Lower abdominal pain,  Fever | APPENDICITIS (acute) | GASTROENTERITIS | GALLSTONES |
| **120** | 29 | Female | 3 hours | Lower abdominal pain,  Left abdominal pain,  Fever | ECTOPIC PREGNANCY | TORSION OF OVARIAN CYST | APPENDICITIS |
| **121** | 78 | Female | 1 week | Constipation | ACUTE BOWEL OBSTRUCTION | BOWEL PERFORATION | DIVERTICULITIS |
| **122** | 40 | Female | 6 weeks | Upper abdominal pain,  Right sided abdominal pain,  Abdominal pain after eating dairy or gluten | GALLSTONES (biliary colic) | GASTRIC ULCER (gastritis) | APPENDICITIS |
| **123** | 21 | Male | 24 hours | Right testicle pain,  Worsening testicle pain | ACUTE TESTICULAR TORSION (torsion testis/appendage) | EPIDIDYMITIS (epididymo-orchitis) | ORCHITIS |
| **124** | 72 | Female | 1 day | Right hip pain,  Recent injury | FRACTURED HIP (neck of femur) | SEPTIC ARTHRITIS | GOUT |
| **125** | 40 | Male | 6 weeks | Left groin pain,  Left groin lump after exercise | INGUINAL HERNIA | BOWEL OBSTRUCTION | DIVERTICULITIS |
| **126** | 50 | Male | 2 weeks | Blood in urine | BLADDER CANCER | RENAL CANCER | UTI |
| **127** | 60 | Female | 2 days | Severe left eye pain | ACUTE GLAUCOMA | UVEITIS | KERATITIS |
| **128** | 30 | Male | 1 day | Eye pain,  Injury | EXTERNAL ORBITAL BRUISING (contusion injury of the eye) | GLOBE RUPTURE | CORNEAL ABRASION |
| **129** | 25 | Male | 2 weeks | Severe mouth ulcers,  Loss of appetite | CROHN'S DISEASE (inflammatory bowel disease) | HERPES INFECTION (stomatitis) | SEVERE APHTHOUS ULCERATION |
| **130** | 69 | Female | 2 weeks | White streaks on tongue | ORAL THRUSH (candida) | LICHEN (planus/sclerosis) | LEUKOPLAKIA (oral hairy) |
| **131** | 23 | Male | 2 months | Discoloured tongue,  Patterns on tongue | GEOGRAPHIC TONGUE | ORAL THRUSH (candida) | POOR ORAL HYGIENE |
| **132** | 40 | Female | 6 months | Lost sense of taste | SMOKING RELATED LOSS OF TASTE (disturbance) | POOR ORAL HYGIENE | B12 DEFICIENCY |
| **133** | 40 | Female | 2 weeks | Lump in left breast | BREAST CANCER | FIBROADENOMA (breast) | BREAST (benign) CYST |
| **134** | 65 | Female | 3 days | Pain in right breast,  Fever | BREAST CANCER (inflammatory) | MASTITIS | CELLULITIS |
| **135** | 28 | Female | 1 month | Rash on face,  Painful rash on legs | LUPUS (systemic lupus erythematosus) | MENINGITIS (infection) | VASCULITIS |
| **137** | 60 | Male | Few hours | Fever,  Dry cough,  Shortness of breath | PULMONARY EMBOLISM | PNEUMONIA | INFLUENZA (viral infection) |
| **138** | 75 | Female | 5 days | Red skin,  Painful skin,  Cut leg | CELLULITIS (due to infected laceration) | NECTORISING FASCIITIS | INFECTED WOUND (cut) |
| **139** | 30 | Male | 1 day | Headache,  Recent injury | SUBDURAL HAEMATOMA (extradural haematoma) | MINOR HEAD INJURY (soft tissue injury/alcohol related injury) | ALCOHOL ABUSE |

### Table S2: Cases for assessment of OSC comprehension

| **Nr** | **Free text chief complaint(s)** | **Standardised chief complaint(s)** | **Single Symptom** | **Variation 1**  **(NLP)** | **Variation 1 (drop-down)** | **Variation 2**  **(NLP)** | **Variation 2 (drop-down)** | **Variation 3**  **(NLP)** | **Variation 3**  **(drop-down)** |
| --- | --- | --- | --- | --- | --- | --- | --- | --- | --- |
| 1 | A 22 year old woman who has had pain in the upper part of her tummy for 6 weeks, after eating. | Upper abdominal pain,  Abdominal pain worse after eating | Upper abdominal pain worse after eating | I have had pain in the upper part of my tummy after eating | upper tummy pain after eating | The top of my belly really hurts me after food | top of belly hurts after food | I have tummy pain at the top after eating | tummy pain at the top after eating |
| 2 | A 18 year old girl has tummy pain low down and is weeing more often than normal for the last 2 days. | Lower abdominal pain,  Frequent urination | Lower abdominal pain | I am having tummy pain lower down | having tummy pain lower down | My tummy hurts below my belly button | tummy pain below bellly button | pain at the bottom of my tummy | pain at the bottom of tummy |
|  |  |  | Frequent urination | I pee a lot | peeing lot | I have to wee all the time | weeing all the time | urinating too much | urinating too much |
| 3 | 19 year old woman  She felt faint and collapsed yesterday. | Feeling faint,  Collapse | Feeling faint | I feel faint | Feeling faint | I feel like I might pass out | Feel like i will pass out | I feel woozy | Feeling woozy |
|  |  |  | Collapsed | I collapsed | collapse | I passed out | Passed-out | I blacked out | blacked out |
| 4 | An 80 year old woman is worried about her memory that has become worse over the last year. | Memory loss | Memory loss | I keep forgetting everything | forgetting everything | I don't remember anything | not remembering | I'm getting absent-minded | absent-minded |
| 5 | A 40 year old man has been experiencing difficulty sleeping as a result of nightmares for over two months. | Having nightmares,Insomnia | Having nightmares | I have horrible nightmares | horrible nightmares | I have bad dreams | bad dreams | I have night terrors | night terrors |
|  |  |  | Insomnia | I cannot sleep | cannot sleep | I have insomnia | insomnia | I have sleeplessness | sleeplessness |
| 6 | A 70 year old retired male farm labourer has left hip pain that is now stopping him from walking the dogs as usual every morning for the last six weeks. | Left hip pain,  Difficulty walking | Left hip pain | My left hip hurts | hurting left hip | Pain in left hip | left hip pain | I have discomfort in my left hip | left hip discomfort |
|  |  |  | Difficulty walking | I cannot walk | cannot walk | I have difficulty walking | difficulty walking | I find it hard to walk | hard to walk |
| 7 | A 30 year old man noticed left knee pain after playing football one week ago.  After the match, his knee became swollen. | Left knee pain after exercise,  Left knee swelling after exercise | Left knee pain after exercise | My left knee hurts after playing football | hurting left knee after playing football | I have pain in left knee after exercise | left knee pain after exercise | I have discomfort in left knee after exercise | left knee discomfort after exercise |
|  |  |  | Left knee swelling after exercise | My left knee swells up after playing football | swollen left knee after playing football | my left knee enlarges after exercise | left knee enlarges after exercise | my left knee bulges after exercise | left knee bulges after exercise |
| 8 | A 60 year old man has right heel pain a month after a walking holiday. | right heel pain after walking | right heel pain after walking | I get right heel pain after walking | right heel pain after walking | My right heel hurts when I walk | hurting right heel when walking | discomfort in right heel after exercise | right heel discomfort after exercise |
| 9 | A 20 year old male care assistant has pain in his lower back after lifting a patient awkwardly one week ago. | lower back pain after heavy lifting | lower back pain after heavy lifting | My lower back hurts after lifting a heavy box | lower back hurting after lifting heavy box | I have lower back pain after lifting a heavy box | lower back pain after lifting a heavy box | I have lower back discomfort after lifting a heavy box | lower back discomfort after lifting a heavy box |
| 10 | A 19 year old male student with pain on the left side of their tongue for two days. | Left sided tongue pain | Left sided tongue pain | The left side of my tongue hurts | left side of tongue hurts | Discomfort on left side of tongue | left side of tongue discomfort | Pain on left side of tongue | left sided tongue pain |
| 11 | 52 year old man.....He has been complaining of stinging when passing urine for the last week. | Painful urination | Painful urination | It is painful when I pee | painful peeing | I have discomfort when I wee | discomfort when weeing | I have burning urine | burning urine |
| 12 | A 72 years old woman.  She has had a one sided headache for 2 days that has kept her awake at night. | One sided headache,  Unable to sleep due to headache | One sided headache | My head hurts on one side | head hurts on one side | Half my head hurts | half of head hurts | Headache on one side | Headache on one side |
|  |  |  | Unable to sleep due to headache | My headache stops me sleeping | headache stops sleep | I have insomnia because of a headache | insomnia because of a headache | My headache keeps me awake | Headache keeps me awake |
| 13 | A 32 year old woman.  Over the last month she has noticed some crampy tummy pains especially when she goes out for pizza. | Crampy abdominal pains,  Abdominal pain after eating dairy or gluten | Crampy abdominal pains | My tummy is cramping | tummy cramping | I have colicky abdominal pain | colicky abdominal pain | I have belly cramps | belly cramps |
|  |  |  | Abdominal pain after eating dairy or gluten | I have tummy pain after eating after dairy or gluten | tummy pain after after dairy or gluten | I have stomach pains worse after dairy or gluten | stomach pains worse after dairy or gluten | I have gut pains worse after dairy or gluten | gut pains worse after dairy or gluten |
| 14 | A 17 year old girl...  Yesterday she had three nosebleeds that were difficult to stop. Today she has noticed some bruises on her thighs.  She looks very pale. | Recurrent nosebleeds,  Bruises on thigh,  Very pale | Recurrent nosebleeds | I keep having a nosebleed | keep having nose bleed | I have recurrent nose bleeds | recurrent nose bleeds | My nose regularly bleeds | regular nose bleeds |
|  |  |  | Bruises on thigh | I have blue and black marks on my thigh | blue and black marks on my thigh | I have a bruise on my thigh | bruises on thigh | I have a contusion on my thigh | contusion on my thigh |
|  |  |  | Very pale | I am white as a sheet | white as sheet | I look anaemic | look anaemic | I look pale | look pale |
| 15 | A 34 year old man who is usually well.  He has had sore glands in his neck for about 2 weeks.  He does not fell particularly unwell but he has been very hot and sweaty at night. | Sore neck glands,  Hot and sweating at night | Sore neck glands | I have painful neck glands | painful neck glands | I have tender neck glands | tender neck glands | I have sore neck glands | sore neck glands |
|  |  |  | Hot and sweating at night | I feel hot at night and perspire | feeling hot at night and perspire | I have night sweats | night sweats | I am hot and sweaty at night | hot and sweaty at night |
| 16 | A 48 year old man....  Over the last week he has developed an aching pain in his lower back. | Lower back pain | Lower back pain | My lower back hurts | lower back hurts | I have lower back pain | lower back pain | I have lower back discomfort | lower back discomfort |
| 17 | A 23 year old man.  He is a bit out of breath and is running a temperature of 38.2 degrees C.  Yesterday morning he accidentally cut his shin deeply and the wound looks hot and angry. | Short of breath,  Fever of 38.2 degrees,  Red wound,  Hot wound | Short of breath | I am running out of breath | running out of breath | I have slight difficulty breathing | slight difficulty breathing | I feel short of breath | short of breath |
|  |  |  | Fever of 38.2 degrees | I have a temperature of 38.2 degrees | temperature of 38.2 degrees | My body temperature is high | high body temperature | I have a fever of 38.2 | fever of 38.2 |
|  |  |  | Red wound | I have a cut with redness around it | cut with redness around it | My cut is inflamed | cut is inflamed | I have a cut that has become red | red cut |
|  |  |  | Hot wound | Warmth around cut | warmth around cut | My wound is warm to touch | warm to touch | The cut feels hot | cut feels hot |
| 18 | A 18 year old girl who has had a 'cold' with a runny nose and watery eyes for two days.  Her eyes are a bit sticky in the morning and watery during the day | Runny nose,  Watery eyes,  Sticky eyes | Runny nose | I am snuffly | snuffly | My nose is watering | watery nose | I have a runny nose | runny nose |
|  |  |  | Watery eyes | I am tearing | tearing | My eyes are weeping | weeping eyes | My eyes are watering | watery eyes |
|  |  |  | Sticky eyes | My eyes stuck together | eyes stuck together | my eyes are gluey | eyes are gluey | I have crusty eyes | crusty eyes |
| 19 | A 55 year old man....For the last 24 hours, he has noticed his urine seems to have a red tinge to it. | blood in urine | blood in urine | My pee is pinkish | pee pinkish | There is blood in my wee | blood in wee | My wee is red | red wee |
| 20 | She has stinging when she goes for a wee, for the last 2 weeks. | Burning pain on urinating | Burning pain on urinating | I feel burning when peeing | burning when peeing | It is painful when I wee | painful when I wee | My wee stings | stinging wee |
| 21 | A 21 year old woman with smelly watery discharge that smells, for 5 weeks. | smelly, watery vaginal discharge | smelly, watery vaginal discharge | Something smelly and watery coming out of my pussy | smelly and water from the pussy | I have smelly, leaking liquid from down below | smelly, leaking liquid from down below | stinky, watery discharge from my vagina | stinky watery vaginal discharge |
| 22 | A 21 year old woman with mild pain in her bladder area for six months.  This has occurred before and after her period. | Pelvic pain before and after period | Pelvic pain before and after period | My lower tummy hurts around my period | lower tummy hurts around period | I have pain at the bottom of my tummy when I menstruate | pain at the bottom of my tummy when I menstruate | lower belly pain around my period | lower belly pain around period |
| 23 | A 24 year old woman.  Her periods have been heavier and more painful for the last six months. | Heavy periods,  Painful periods | Heavy periods | my menstrual bleeding is heavy | menstrual bleeding heavy | I am passing clots during my period | passing clots during my period | I have heavy periods | heavy periods |
|  |  |  | Painful periods | my menstrual bleeding is painful | menstrual bleeding painful | it hurts a lot during my time of the month | hurts a lot during time of the month | my periods really hurt | periods really hurt |
| 24 | A 52 year old woman has sudden upper tummy pain.  The pain now feels like it is also in her chest | Upper abdominal pain,  Chest pain | Upper abdominal pain | I am having tummy pain higher up | tummy pain higher up | My tummy hurts above my belly button | tummy pain above belly button | pain at the top of my tummy | pain at the top of tummy |
|  |  |  | Chest pain | the upper part of my torso is painful | upper torso painful | I have discomfort in my chest | discomfort in my chest | It hurts around my chest | chest hurts |
| 25 | An 85 year old man has sudden pain in the middle of his tummy.  In the last hour, the pain is worse, and is now going through to his back and groin. | Central abdominal pain,  Abdominal pain radiating to back and groin | Central abdominal pain | I am having tummy pain around my umbilicus | tummy pain around my umbilicus | My tummy hurts around my belly button | tummy pain around belly button | I have pain at the centre my tummy | pain at the centre my tummy |
|  |  |  | Abdominal pain radiating to back and groin | I have tummy pain going to the back and groin | tummy pain to the back and groin | I have abdominal pain that spreads to my back and crotch | abdominal pain that spreads to my back and crotch | my belly ache spreads towards my back and balls | belly ache spreads towards my back and balls |
| 26 | A 28 year old woman has right-sided tummy pain which has got worse throughout the day.  She feels sick, and has not managed to eat since breakfast. | Right sided abdominal pain,  Nausea,  Loss of appetite | Right sided abdominal pain | I am having tummy pain on the right | tummy pain on the right | My tummy hurts on the right side | tummy hurts on the right side | I have pain on the right of my tummy | pain on the right of my tummy |
|  |  |  | Nausea | I feel nauseous | feeling nauseous | I am feeling queasy | feeling queasy | I feel like I want to vomit | feeling like you want to vomit |
|  |  |  | Loss of appetite | I am off my food | off my food | I have a poor appetite | poor appetite | I don't feel like eating | don't feel like eating |
| 27 | A 60 year old man has had chest tightness for the last month, and the pain moves into his neck whenever he walks uphill. | Chest tightness,  Chest pain worse on exertion,  Chest pain radiating to neck | Chest tightness | My chest feels tight | chest feels tight | my chest feels constricted | chest feels constricted | I feel pressure on my chest | pressure on my chest |
|  |  |  | Chest pain worse on exertion | I have a painful chest when doing anything | painful chest when doing anything | I have chest discomfort when i walk | chest discomfort on walking | I have chest pain on walking | chest pain on walking |
|  |  |  | Chest pain radiating to neck | I have chest pain that moves into my neck | chest pain moving into neck | I have chest discomfort that travels to my neck | chest discomfort that travels to my neck | My chest hurts that spreads to my neck | chest hurts spreading to neck |
| 28 | A 36 year old man who has a fast heartbeat a few times a week over the last 2 months. | Palpitations | Palpitations | My heart races | heart races | My heart is beating fast | heart is beating fast | My heart is pounding | heart is pounding |
| 29 | A 47 year old man who sees things floating in his left eye.  This has happened every now and again but over the last week or so he has noticed more. | Eye floaters | Eye floaters | I see things are floating in my eyes | objects floating in the eye | I see dark dots in my eyes | dark dots in my eyes | I can see squiggly lines | see squiggly lines |
