# Supplementary material for: Controlling Inputter Variability in Vignette Studies Assessing Web-Based Symptom Checkers: Evaluation of Current Practice and Recommendations for Isolated Accuracy Metrics
Source: JMIR Form Res. 2024 May 31;8:e49907. doi: 10.2196/49907 (PMC11179013; doi:10.2196/49907)
Supplement: Multimedia Appendix 2 [file formative_v8i1e49907_app2.docx]

|  | Free input | Partially free testers | Restricted testers |
| --- | --- | --- | --- |
| Chief complaint(s) | - Chosen and phrased freely by tester - If not accepted by OSC, vignette entry stopped | - Chief complaint(s) prescribed and inputted as described - If not accepted by OSC, vignette entry stopped | - Chief complaint(s) prescribed and inputted as described - If not accepted by OSC, vignette entry stopped |
| Order of symptom entry | - Chosen in the order as requested by OSC after chief complaint(s) entry | - Chosen in the order as requested by OSC after chief complaint(s) entry | - Chosen in the order as requested by OSC after chief complaint(s) entry |
| Instruction for answering questions during the question-answer part of the consultation | - To select symptom only if mentioned in vignette - To decline symptoms unless specifically mentioned in the vignette. However, if testers felt a symptom could be true for a real case but is missing from the vignette they were to select that symptom - If symptoms of chief complaint(s) not recognised but asked later to select it | - To select symptom only if mentioned in vignette - To decline symptoms unless specifically mentioned in the vignette. However, if testers felt a symptom could be true for a real case and the symptom is a “logical extension” of one symptom resulting from another and missing from the case they were to select that symptom - If symptoms of chief complaint(s) not recognised but asked later to select it | - To select symptom only if mentioned in vignette - Not to select symptom even if tester felt it might be missing from the vignette - If synonyms offered for symptom described in the vignette to select it e.g. “abdominal pain” for “tummy pain” - If a bigger category of a symptom offered to select it e.g. “knee pain” in vignette, “leg pain” - If the sentence in the vignette is worded differently but has the same meaning to select it. - If a sentence in a vignette indirectly could lead to a symptom or vice versa, then not to select that symptom. E.g. if someone is 'waking up at night with pain', but the symptom checker asks if the 'pain is severe' - not to select that symptom as i is unknown whether the symptom is severe, just they are waking up at night with it   However, if vignette states that someone is 'crying with the pain' then to select as it can be understood as a ”synonym” the 'pain is severe'   - If symptoms of chief complaint(s) not recognised but asked later to select it |
| Documentation | - Free text entry and other entered symptom via drop-down - Confirmed symptoms of chief complaint(s) - Outcome conditions - Triage advice - All the data points (including confirmed and declined symptoms) | - Free text entry and other entered symptom via drop-down - Confirmed symptoms of chief complaint(s) - Outcome conditions - Triage advice - All the data points (including confirmed and declined symptoms) | - Free text entry and other entered symptom via drop-down - Confirmed symptoms of chief complaint(s) - Outcome conditions - Triage advice - All the data points (including confirmed and declined symptoms) |
