# Supplementary material for: Controlling Inputter Variability in Vignette Studies Assessing Web-Based Symptom Checkers: Evaluation of Current Practice and Recommendations for Isolated Accuracy Metrics
Source: JMIR Form Res. 2024 May 31;8:e49907. doi: 10.2196/49907 (PMC11179013; doi:10.2196/49907)
Supplement: Multimedia Appendix 3 [file formative_v8i1e49907_app3.docx]

##

| **Age**  (Min - Max)  Average | 17 - 85  41.2 |
| --- | --- |
| **Age distribution**  15-19  20-24  25-29  30-34  35-39  40-44  45-49  50-54  55-59  60-64  65-69  70-74  75-79  80-84  85-89  90+ | 12 (10.5%)  18 (15.8%)  13 (11.4%)  10 (8.8%)  6 (5.3%)  8 (7%)  8 (7%)  9 (7.9%)  4 (3.5%)  5 (4.4%)  9 (7.9%)  4 (3.5%)  6 (5.3%)  1 (0.9%)  1 (0.9%)  0 (0%) |
| **Male: female ratio** | 57:53 |
| **Duration of symptoms**  <1 hours  1h - 1 day  More than 1 day - 1 week  More than 1 week - 1 month  More than 1 month - 1 year  1 year < | 3 (2.6%)  22 (19.3%)  30 (26.3%)  29 (25.4%)  30 26.3%)  0 (0%) |
| **Expected triage**  Emergency - Ambulance  Emergency - AE  Urgent - 12h  Urgent - 48h  Routine  Selfcare  Self-limiting | 10 (8.8%)  14 (12.3%)  22 (19.3%)  26 (22.8%)  22 (19.3%)  11 (9.65%)  9 (7.8%) |
| **Medical domains of expected outcome conditions (ICD-11)***  01 Certain infectious or parasitic diseases  02 Neoplasms  03 Diseases of the blood or blood-forming organs  04 Diseases of the immune system  05 Endocrine, nutritional or metabolic diseases  06 Mental, behavioural or neurodevelopmental disorders  08 Diseases of the nervous system  09 Diseases of the visual system  10 Diseases of the ear or mastoid process  11 Diseases of the circulatory system  12 Diseases of the respiratory system  13 Diseases of the digestive system  14 Diseases of the skin  15 Diseases of the musculoskeletal system or connective tissue  16 Diseases of the genitourinary system  18 Pregnancy, childbirth or the puerperium  22 Injury, poisoning or certain other consequences of external causes  24 Factors influencing health status or contact with health services | 32 (9.4%)  24 (7.1%)  3 (0.9%)  6 (1.8%)  5 (1.5%)  21 (6.2%)  32 (9.4%)  12 (3.5%)  4 (1.2%)  22 (6.5%)  25 (7.4%)  41 (12.1%)  4 (1.2%)  44 (13%)  36 (10.6%)  4 (1.2%)  21 (6.2%)  3 (0.9%) |

*For 12 of the expected outcome conditions no ICD11 domain could be found, hence assigned by the research team
