# Supplementary material for: Controlling Inputter Variability in Vignette Studies Assessing Web-Based Symptom Checkers: Evaluation of Current Practice and Recommendations for Isolated Accuracy Metrics
Source: JMIR Form Res. 2024 May 31;8:e49907. doi: 10.2196/49907 (PMC11179013; doi:10.2196/49907)
Supplement: Multimedia Appendix 4 [file formative_v8i1e49907_app4.docx]

| **Identifier** | **Gender** | **Age** | **Qualification** | **Study phase** |
| --- | --- | --- | --- | --- |
| Tester 1 | M | 24 | In university | Free tester, repeat free tester, partially free tester |
| Tester 2 | M | 21 | In university | Free tester, repeat free tester |
| Tester 3 | M | 38 | BA | Free tester, repeat free tester, restricted tester, repeat of “15 failed test cases” |
| Tester 4 | F | 30 | Diploma | Partially free tester, restricted tester |
| Tester 5 | F | 39 | BA, MBA | Partially free tester, restricted tester |
| Tester 6 | M | 24 | In university (medical student) | Tester for comprehension of OSCs |
