# Supplementary material for: Controlling Inputter Variability in Vignette Studies Assessing Web-Based Symptom Checkers: Evaluation of Current Practice and Recommendations for Isolated Accuracy Metrics
Source: JMIR Form Res. 2024 May 31;8:e49907. doi: 10.2196/49907 (PMC11179013; doi:10.2196/49907)
Supplement: Multimedia Appendix 5 [file formative_v8i1e49907_app5.docx]

|  | Accuracy of outcome condition |
| --- | --- |
| **Free group** |  |
| Tester 3 | 41.2% |
| Tester 1 | 51.8% |
| Tester 2 | 43.9% |
| Average of 3 testers | 45.6% |
| **Partially free group** |  |
| Tester 4 | 56.1% |
| Tester 5 | 55.3% |
| Tester 1 | 56.1% |
| Average of 3 testers | 55.8% |
| **Restricted group** |  |
| Tester 3 | 50.9% |
| Tester 4 | 57% |
| Tester 5 | 43.9% |
| Average of 3 testers | 50.6% |
