# Supplementary material for: Controlling Inputter Variability in Vignette Studies Assessing Web-Based Symptom Checkers: Evaluation of Current Practice and Recommendations for Isolated Accuracy Metrics
Source: JMIR Form Res. 2024 May 31;8:e49907. doi: 10.2196/49907 (PMC11179013; doi:10.2196/49907)
Supplement: Multimedia Appendix 6 [file formative_v8i1e49907_app6.docx]

|  | N of vignettes affected |
| --- | --- |
| Symptoms missed completely | 39 |
| Symptom missed as wider logical category | 17 |
| Additional symptoms added wrongly | 33 |
| Different interpretation of symptoms | 10 |
| Error with duration | 4 |
| Incompatibility between OSC function and instructions | 6 |

Analysing the cases of the restricted group where vignettes were not followed perfectly showed that there were 39 cases where symptom(s) in the consultation were missed completely, 17 cases had a missing symptom of a wider logical category, 33 cases had symptoms not described in the vignette (some cases had multiple issues and/or the same issue repeated multiple times and/or by multiple testers). In 10 cases the vignettes symptoms were misinterpreted (this could be due to the Healthily OSC being unclear in expressing offered symptoms or the user being unsure of the original symptom meaning), 3 cases had errors with the duration of how long the symptom had been present, in 6 cases the instructions given to the testers did not comply with how the Healthily OSC works
