# Supplementary material for: Controlling Inputter Variability in Vignette Studies Assessing Web-Based Symptom Checkers: Evaluation of Current Practice and Recommendations for Isolated Accuracy Metrics
Source: JMIR Form Res. 2024 May 31;8:e49907. doi: 10.2196/49907 (PMC11179013; doi:10.2196/49907)
Supplement: Multimedia Appendix 7 [file formative_v8i1e49907_app7.docx]

| Chief complaint(s) | - Copy and paste the prescribed chief complaint(s) word by word (if only one symptom can be added at the time, add one by one) - If none of the symptoms are accepted by OSC, stop the vignette entry - In case of multiple chief complaints, if at least one of the symptoms is successfully inputted, continue the consultation. - If not all the symptoms are successfully inputted but there is an option to add more, attempt to do so. If unsuccessful, continue with the consultation without adding them. |
| --- | --- |
| Demographics | - Copy over/input age and gender of the persona of the vignette |
| Instruction for answering questions during the question-answer part of the consultation | - Only select symptoms that are mentioned in the vignette - Do not to select any symptom that is not described in the vignette even if you feel it might be missing from the vignette - Caveats:   - If synonyms offered for symptom described in the vignette, select it e.g. “abdominal pain” for “tummy pain”   - If a wider logical category of a symptom is offered, select it e.g. if “knee pain” is in the vignette, then select “leg pain” if offered   - If the sentence in the vignette is worded differently but has the same meaning, select it.   - If a sentence in a vignette indirectly could lead to a symptom or vice versa, then do not select that symptom. E.g. if someone is 'waking up at night with pain', but the symptom checker asks if the 'pain is severe' - do not select that symptom as it is unknown whether the symptom is severe, just they are waking up at night with it. However, if the vignette states that someone is 'crying with the pain' then you can select it, as it may be understood as a ”synonym” of 'pain is severe'   - If further characteristics of a confirmed symptom is asked in one question with multiple answers, and there is an option to decline all of them, do so. If this is not possible but there is an option not to answer e.g. “I do not know”/”Not prefer to say”, choose that, otherwise, stop the consultation and mark the case as “INCOMPLETE” - If some of the chief complaint(s) are not recognised but asked later during the consultation, confirm them |
| Documentation | - Free text entry and other entered symptoms via drop-down - Confirmed chief complaint(s) - Outcome conditions - Triage advice - All the data points (including confirmed and declined symptoms) - depending on symptom checker, if possible copy the consultation report with as much detail as possible |
